# Asynchronicity of Facial Blood Perfusion in Migraine

**DOI:** 10.1371/journal.pone.0080189

**Published:** 2013-12-04

**Authors:** Nina Zaproudina, Victor Teplov, Ervin Nippolainen, Jukka A. Lipponen, Alexei A. Kamshilin, Matti Närhi, Pasi A. Karjalainen, Rashid Giniatullin

**Affiliations:** 1 Institute of Biomedicine, University of Eastern Finland, Kuopio, Finland; 2 Institute of Dentistry, University of Eastern Finland, Kuopio, Finland; 3 Department of Applied Physics, University of Eastern Finland, Kuopio, Finland; 4 Department of Neurobiology, A. I. Virtanen Institute, University of Eastern Finland, Kuopio, Finland; Charité University Medicine Berlin, Germany

## Abstract

Asymmetrical changes in blood perfusion and asynchronous blood supply to head tissues likely contribute to migraine pathophysiology. Imaging was widely used in order to understand hemodynamic variations in migraine. However, mapping of blood pulsations in the face of migraineurs has not been performed so far. We used the Blood Pulsation Imaging (BPI) technique, which was recently developed in our group, to establish whether 2D-imaging of blood pulsations parameters can reveal new biomarkers of migraine. BPI characteristics were measured in migraineurs during the attack-free interval and compared to healthy subjects with and without a family history of migraine. We found a novel phenomenon of transverse waves of facial blood perfusion in migraineurs in contrast to healthy subjects who showed synchronous blood delivery to both sides of the face. Moreover, the amplitude of blood pulsations was symmetrically distributed over the face of healthy subjects, but asymmetrically in migraineurs and subjects with a family history of migraine. In the migraine patients we found a remarkable correlation between the side of unilateral headache and the direction of the blood perfusion wave. Our data suggest that migraine is associated with lateralization of blood perfusion and asynchronous blood pulsations in the facial area, which could be due to essential dysfunction of the autonomic vascular control in the face. These findings may further enhance our understanding of migraine pathophysiology and suggest new easily available biomarkers of this pathology.

## Introduction

Migraine is a common neurological disorder with complex and still essentially unknown mechanisms [1], [2]. The contribution of the vasculature to migraine pathology was suggested in early studies of this disease [3] but still remains controversial [4]–[9]. One typical criterion of migraine is the unilateral character of the headache [10]. Such lateralization suggests potential asymmetrical function also in the vascular bed including extracranial vessels. To date, the asymmetrical vascular changes in migraine have been addressed directly only rarely. Classical migraine studies have already noted that frontal branches of the superficial temporal artery may be dilated on the side of the headache [3]. Likewise, possible local asymmetry of forehead blood flow has been reported in the affected side of the migraineurs’ face during an attack [11]. It was also reported that in the interictal period the pupil of the symptomatic side dilated more slowly and less extensively in the darkness than the opposite pupil, indicating an asymmetry of sympathetic outflow [12]. Asymmetry in the sympathetic skin responses was also found in the headache-free period in unilateral migraineurs [13], supporting the impairment of sympathetic control in the face. However, the relative phase of blood pulsations in the face of migraine patients has not been studied so far.

To find new vascular biomarkers of migraine we applied a recently developed system of blood pulsations imaging [14] to map both the amplitude and relative phase of blood pulsations over the subject’s face. The aim of the present study was evaluation and comparison of the magnitude and symmetry of blood pulsation characteristics in the facial area of migraine patients (MP) and healthy subjects with (FH) and without (HS) a family history of migraine.

## Subjects and Methods

### Participants

Our study consisted of 38 women representing three groups: MP group (n  =  13) and 25 healthy subjects with (n  =  8) and without a family history of migraine (n  =  17). To avoid gender-related differences, only women were studied. These groups did not differ as regards to the age or anthropometric characteristics apart the values of diastolic blood pressure (p<0.05, one way ANOVA, Table 1). All subjects were recruited from the University staff and students. They did not have any prior injuries or severe structural deformities in the facial area and were all except one non-smokers. Persons with any neurologic, cardiovascular or skin diseases and those using any vasoactive drugs were excluded. Three subjects suffered from hypothyroidism treated with replacement therapy (two in the HS and one in FH group).

**Table 1:** Characteristics of the subjects under study.

	MP (n = 13)	FH (n = 8)	HS (n = 17)
Age (years)	35.7 (3.1)	32.4 (3.4)	33.8 (2–2)
Height (cm)	166.6 (2.0)	168.9 (1.7)	167.2 (1.6)
Weight (kg)	61.8 (3.7)	62.1 (3.5)	62.9 (2.3)
Handedness (R/L)	11/2	7/1	17/0
Family history of migraine	11/13	8/8	0/17
Systolic blood pressure (mm Hg)	116.1 (3.8)	109.9 (3.4)	110.9 (2.2)
Diastolic blood pressure (mm Hg)	82.1 (3.1)^ #^	72.8 (2.8)^ #^	74.1 (2.0)^ #^
Heart rate (beats/min)	69.7 (2.5)	65.0 (3.9)	63.6 (2.3)
Menses day	17.7 (3.6)	15.2 (4.1)	18.3 (3.0)

Data are mean values (standard error of the mean); ^#^p<0.05 between the groups.

All subjects of the MP group fulfilled the diagnostic criteria of migraine according to the International Classification of Headache Disorders (2004) [10], and six of them had migraine with aura. The headache was restricted to the right side in six, to the left in three, and was without clear laterality in four subjects. During attacks, vomiting or nausea presented in nine subjects, and photo- and phonophobia in all subjects. Four migraineurs used anti-migraine medications to prevent attacks. A considerably higher proportion of the migraineurs (84.6%) reported a family history of migraine than the healthy subjects (32%, p<0.005, Fisher’s Exact test). The main characteristics of subjects under study are presented in Table 1.

### Ethics statement

This study was conducted in accordance with the ethical standards laid down in the 1964 Declaration of Helsinki. The study plan was approved on 24.01.2012 by the Research Ethics Committee, Hospital District of Northern Savo. All subjects provided a written consent and completed a standard questionnaire.

### Perfusion measurements

**Figure 1:**
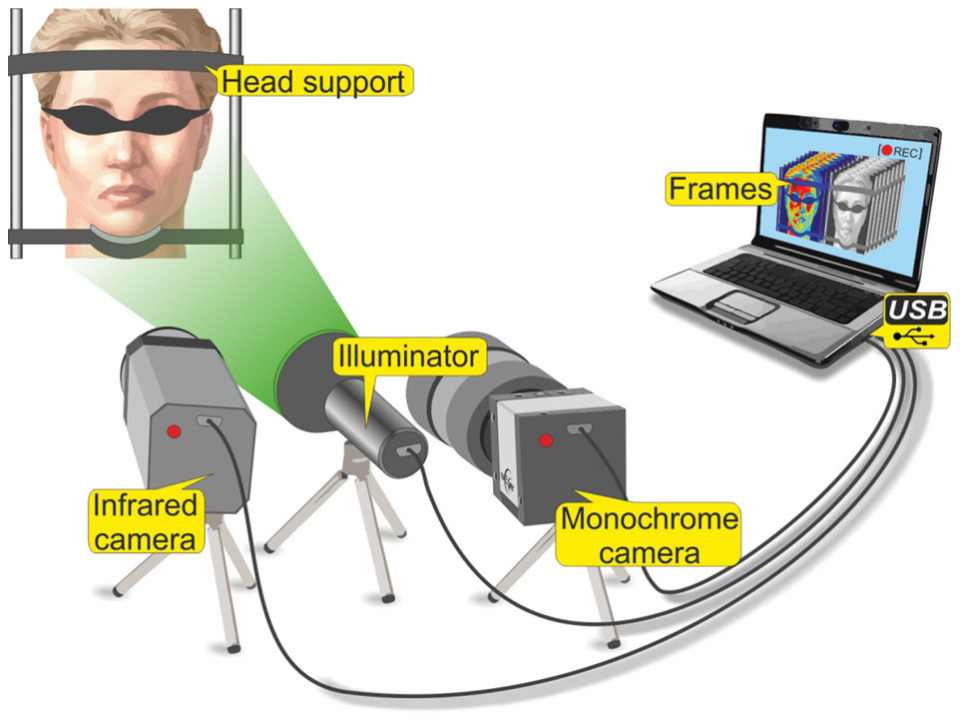
The experimental setup for recording of the blood perfusion in the facial area.

The setup for carrying out experiments is shown in Fig. 1. In our experiments, the facial area of a subject was illuminated by an illumination system which consists of two similar light-emitting diodes (LED) of the model H2A3-530 by Roithner LaserTechnick, operating at a wavelength of 530 nm. The optical power of both LEDs was 60 mW, providing an illuminated area of 30×60 cm^2^ at a distance between the subject face and video cameras, which was about 2 m. The duration of each measurement was 15 s. The illuminating light was linearly polarized. The direction of illumination was at a small angle with the direction of observation provided by the video systems. For video recording of the light reflected from the subject’s face we simultaneously used two video systems: a digital monochrome camera for blood pulsation imaging and a digital infrared camera for thermography imaging. A digital infrared camera system (FLIR A315, FLIR Systems Inc., USA) was used for thermography imaging. Parameters of the infrared camera system during recording were the following: spatial resolution 320×240 pixels, temperature accuracy measurement 0.05 °C, and temporal resolution 9 Hz.

For the blood pulsation imaging (BPI) measurements we used a digital monochrome camera (8-bit model EO-1312 by Edmund Optics) with camera lens (Canon TV-zoom 18 -108). The polarizer was attached to the camera lens. Its transmission axis was oriented orthogonal to the polarization vector of the illuminating light. By using crossed polarizers it was possible to use the light which contains information about main chromophores in epidermis (melanin) and dermis (haemoglobin) [15], [16]. The spatial resolution of recorded video frames was 460×640 pixels with the temporal resolution of 10 Hz. Recording time for both systems was 15 sec for one measurement.

All measurements were done in an air-conditioned dark laboratory maintained at a temperature of 22–24°C and relative humidity of 30–50%, at the same time of the day in spring-summer 2012. In migraineurs, the measurements were performed in the headache-free period. Eating or drinking during the two hours and significant physical activity on the whole day preceding the tests were not allowed. The subject spent at least fifteen minutes in the laboratory in order to achieve thermo equilibrium and to be prepared for the measurements. During the measurements the eyes of each subject were covered by a light protecting mask. We used a stationary support for the subject’s head to minimize the contribution of motion artifacts to BPI parameters.

### Data analysis

**Figure 2:**
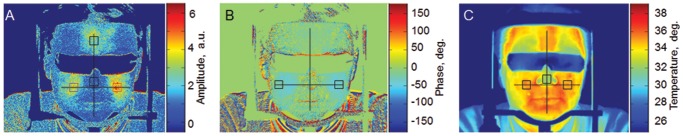
Examples of the images obtained with the BPI system and with the thermographic camera: (A) 2D distribution of the blood pulsation amplitude; (B) 2D map of the blood pulsation phase; (C) 2D distribution of the skin temperature.

All recorded videos from both systems were processed off-line by using custom software implemented in the MATLAB platform. The blood pulsation imaging (BPI) technique [14] was used for 2D mapping of both the blood pulsation amplitude and of the relative phase for the face of each subject. The proposed technique is based on synchronous amplification of those pixel values in the recorded video frames, which vary in time with the current heart-beat rate. A reference function, required for the synchronous detection of cardiovascular pulse waves was formed from the same images. For each image we calculated average pixel values over the whole area of the subject’s face. The result of these averaging was raw photoplethysmography (PPG) signal which is modulated in time. This temporal modulation is caused mainly due to periodical blood volume modulation occurring synchronously with each heartbeat. Further this raw PPG signal was used to generate the complex reference function which is necessary for the synchronous amplification of the recorded series of frames. The result of this amplification is a complex correlation matrix which has the same size as the recorded video frames. Therefore, the modulus of each pixel of this matrix is proportional to the modulation amplitude of blood pulsations while the argument of the pixels value describes the spatial distribution of the blood pulsation phase. It should be mentioned that the correlation matrixes for all subjects under study were calculated after averaging over 15 s to increase the signal-to-noise ratio. Examples of the spatial distribution of the amplitude and relative phase of blood volume pulsation, and of the temperature (thermographic image) for one of the subjects are shown in Fig. 2.

For quantitative estimation of the asymmetry in blood pulsations distributions we used the following algorithm. For estimation of the amplitude asymmetry we used a map of blood pulsation amplitude (Fig. 2A). In this map we first found the area with the highest amplitude in one of the cheeks and placed the region of interest (ROI) in this area (shown by the red square in Fig. 2A). Then we placed the second ROI of the same size in another cheek symmetrically to the median of the face (the grey square in Fig. 2A). The amplitude asymmetry was calculated as the difference in blood pulsations amplitude averaged within two chosen ROIs normalized to the mean value of BP amplitude within both ROIs. In addition we placed the third ROI (not shown in Fig. 2) in the nose area, which allows us to find difference of the amplitude of blood pulsation between the nose and cheeks and to compare it with the similar difference in skin temperature values measured by the thermography camera. Skin temperature values were calculated with a custom made Matlab based software using the similar ROIs as in BPI analysis. The nose-cheek difference was calculated by subtracting the temperature of the nose from the average temperature of the cheeks.

The relative phase of blood pulsations quantitatively indicates any delay in the blood delivery. For estimation of the temporal asymmetry we used the map of BP phase (Fig. 2B). In this case we placed two ROIs on the cheeks symmetrically with respect to the medial line at the longest possible distance from the median but not touching the face’s borders (black squares in Fig. 2B), and calculated the difference of mean BP phase within these ROIs.

The recorded frames of both video systems were processed off-line by using algorithms implemented in the MATLAB® platform as described above. The processing of video frames was done blindly: the researchers responsible for the data analysis did not know which group the subject belonged to.

The statistical analysis of measured data was carried out by using the SPSS Statistical Software 17.0 for Windows (SPSS Inc, Chicago, IL, USA). Calculated BPI amplitude and phase parameters, temperature measurements, and the level of asymmetry and cheek-nose differences in these findings were compared between the groups using the Kruskal-Wallis test with the Mann-Whitney U-tests and Bonferroni correction or with the one-way ANOVA with the Tukey post-hoc test, depending on the normality of distributions of the variables. The comparison of the subjects’ characteristics in the three groups was performed using one-way ANOVA. The Fisher’s exact test was applied to compare the proportions of the existing family history of migraine in the patient and control groups and to analyze the relationship between the direction of the BPI wave and the side of headache in unilateral migraine. The relationship between BPI findings and factors, influencing these parameters, was examined using Spearman correlation analysis. The level p<0.05 was considered as significant.

## Results

### Blood perfusion asymmetry in migraine

**Figure 3:**
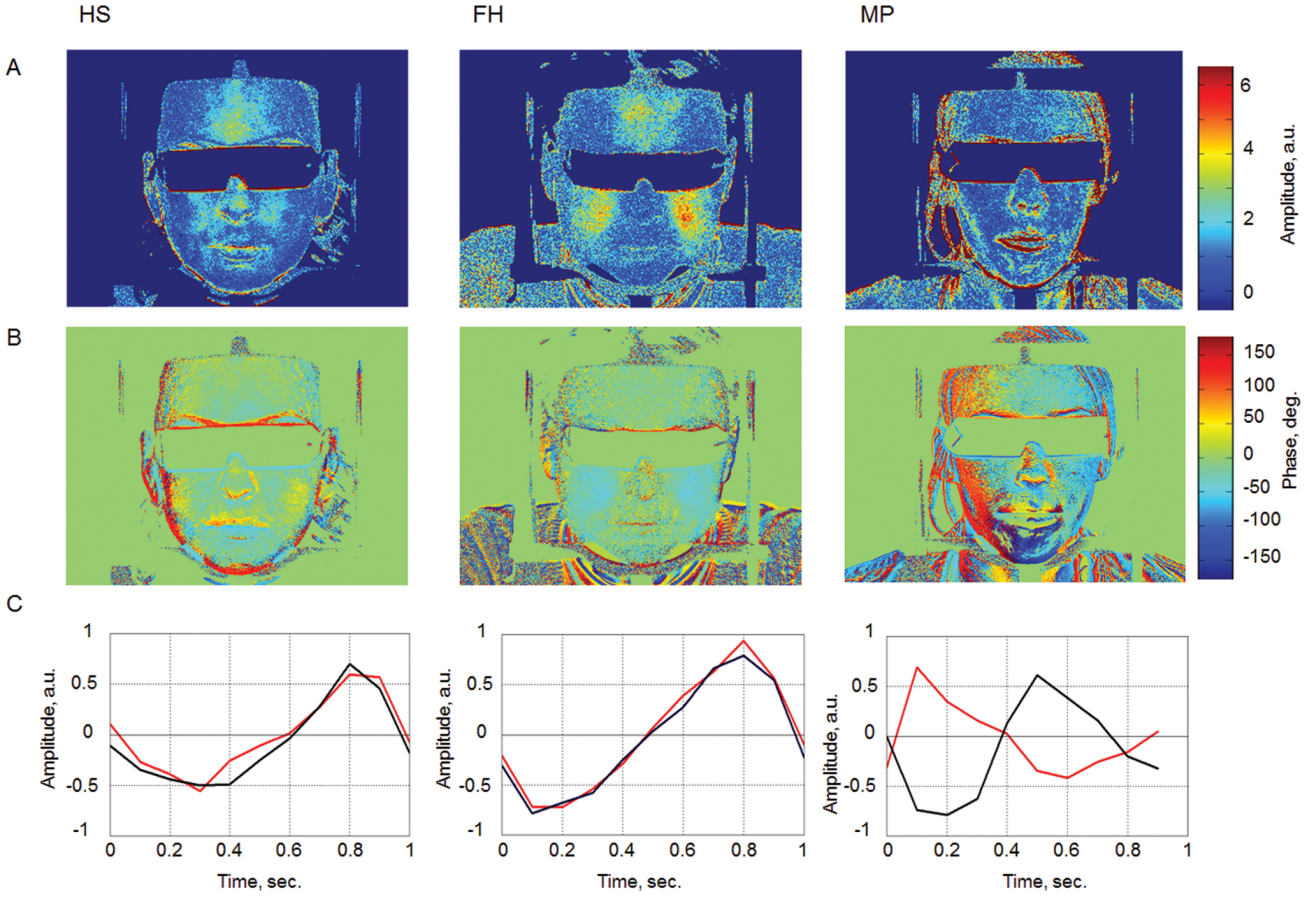
Spatial distribution of facial blood pulsations. Row A, a 2D map of the blood pulsation amplitude; row B, a 2D map of the blood pulsation phase; row C, evolution of the blood pulsation volume during a single cardiac cycle in the right (black line) and left (red line) cheeks of each subject. Column HS: a healthy subject; column FH: a subject from the group with a family history of migraine; column MP: a subject suffering from migraine (left sided).

In the maps of blood pulsations obtained from the facial area, two key parameters of the peripheral blood flow, the blood-pulsation amplitude and blood pulsation phase were determined [14], [17]. Special attention was paid to potential facial asymmetry of these characteristics of blood perfusion in relation to migraine. The algorithm and criteria of such estimations were described in the previous section. Figure 3 shows examples of two-dimensional (2D) distributions of blood pulsation parameters at the facial area of subjects obtained with the BPI technique. The maps of blood pulsation amplitude are shown in row A, while the maps of blood pulsation phase are shown in row B of Fig. 3. To illustrate the influence of the relative phase of blood pulsations, the dynamics of the blood volume change in the right and left cheeks of each subject during a single cardiac cycle is shown in row C. As one can see, the blood volume increases/decreases synchronously in both cheeks in the case of healthy persons (columns HS and FH). In sharp contrast, the blood volume reaches its maximum at different moments in the right and left cheeks of the migraineur (the column MP). The observed asynchronous blood delivery to different parts of the face can be considered a temporal asymmetry of blood perfusion.

Examples of time evolution of the blood volume change during a single cardiac cycle for the respective subjects of Fig. 3A are shown in row C of Fig. 3. It should be emphasized that the curves of Fig. 3C were directly calculated from the raw PPG data (before applying the synchronous amplification technique) of the recorded video frames. These data were obtained by averaging the pixel values within the ROIs of the same size and positions as in Fig. 2B. We show in Fig. 3C the time evolution of the only alternating component of the averaged pixel value during an arbitrarily chosen cardiac cycle, in contrast with the results of Figs. 3A and 3B, where the data were synchronously amplified and time-averaged during 15s.

**Figure 4:**
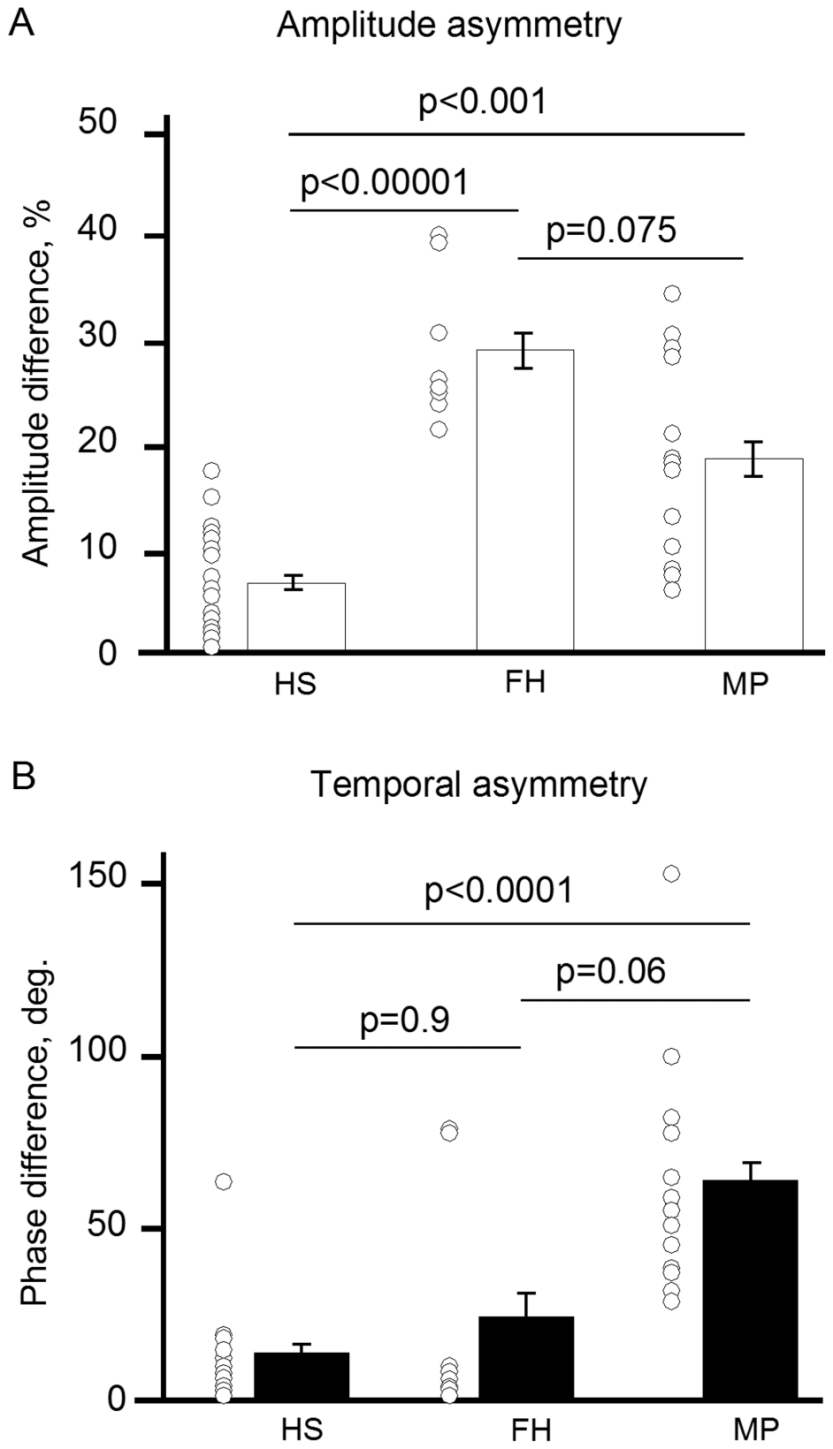
Distribution of the side-to-side difference in blood pulsation parameters among groups of migraine patients (MP) and healthy subjects with (FH) and without (HS) family history of migraine. Bars: mean values with SDs, circles: individual values. A, the amplitude difference, B, the phase difference.

Whereas mean values of the amplitude of blood pulsation did not differ between the groups, the level of asymmetry in the amplitude values was significantly higher in women from both MP and FH groups than in the HS group (p<0.00001 for FH and p<0.001 for MP, Mann-Whitney tests with Bonferroni correction, Fig. 4). The most interesting finding was the strong temporal asymmetry of blood perfusion seen in all women suffering from migraine (p<0.00001 between MP and HS groups, Fig. 4B). Our experiments show a high degree of accuracy in distinguishing between the healthy subjects and migraineurs: the values of sensitivity and specificity, interpreted by an investigator blinded to the study groups, were 61.5% and 100% for the amplitude asymmetry, and 100% and 94.1% for the temporal asymmetry, respectively.

### Transverse wave of blood perfusion

The observed temporal asymmetry or asynchronicity can be shown in complex 2D animations (see Videos S1 and S2) as a novel phenomenon of transverse (side-to-side) waves of blood perfusion in the face of migraineurs. For formation of animations we used the correlation matrix which contains the information about spatial distribution of both the amplitude and phase of blood pulsations. The bipolar imaging technique is used to represent variations of the blood volume during single cardiac cycle. To visualize the dynamics of blood volume pulsations we show in these animations the series of 36 frames in which the distribution of the BP amplitude was calculated for the phase varying sequentially with a step of 10 degrees. The values assigned to the pixel of the frames in the animations can be positive, negative or zero. Positive values represent an increase of the blood volume with respect to its average value, while the negative values show the blood volume decrease. In both videos, the magenta color is assigned to the negative pixel values while the green color is assigned to the positive values. More bright green or magenta corresponds to larger variations of the blood volume while the dark is zero value. One can clearly see the transverse wave in Video S1 for a migraineur whereas this wave is absent in Video S2 for a healthy subject. Subjects shown in Figs. 2, 3 and in Videos S1, S2 have given written informed consent, as outlined in the PLOS consent form, for publications of their photographs.

Unlike in the great majority of the cases of HS and FH groups, the blood was delivered to the face asynchronously in the migraineurs. In the FH group, the temporal asymmetry was found in two of eight women. The mean values tended to differ between FH and MP groups (p  =  0.06, Mann-Whitney tests with Bonferroni correction, Fig. 4B).

### Laterality of headache

**Figure 5:**
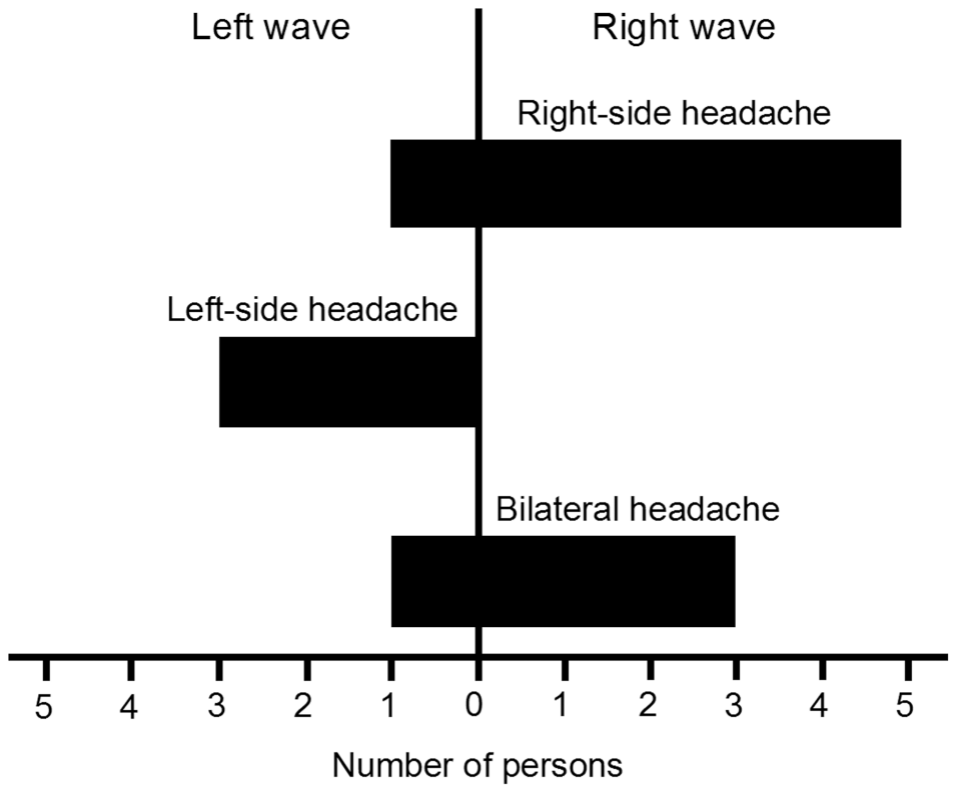
Number of the migraine patients possessing either right or left transverse wave of blood perfusion in relation to the side of the headache.

Apart from significant temporal asymmetry observed in the MP group we found a remarkable correlation between a side of unilateral headache and the direction of the blood perfusion wave. This relation (p<0.05, Fisher’s Exact test) is shown in Fig. 5 where it is evident that the right-to-left wave was mainly observed in migraineurs with right-side headache and vice versa. Thus, in the left-side headache, the blood, within the cardiac cycle, was first delivered to the left side of the face and then, after a delay (ranging from 68 to 425 ms), to the right side, whereas for others it was delivered in the inverse order.

### BPI and thermography findings

**Figure 6:**
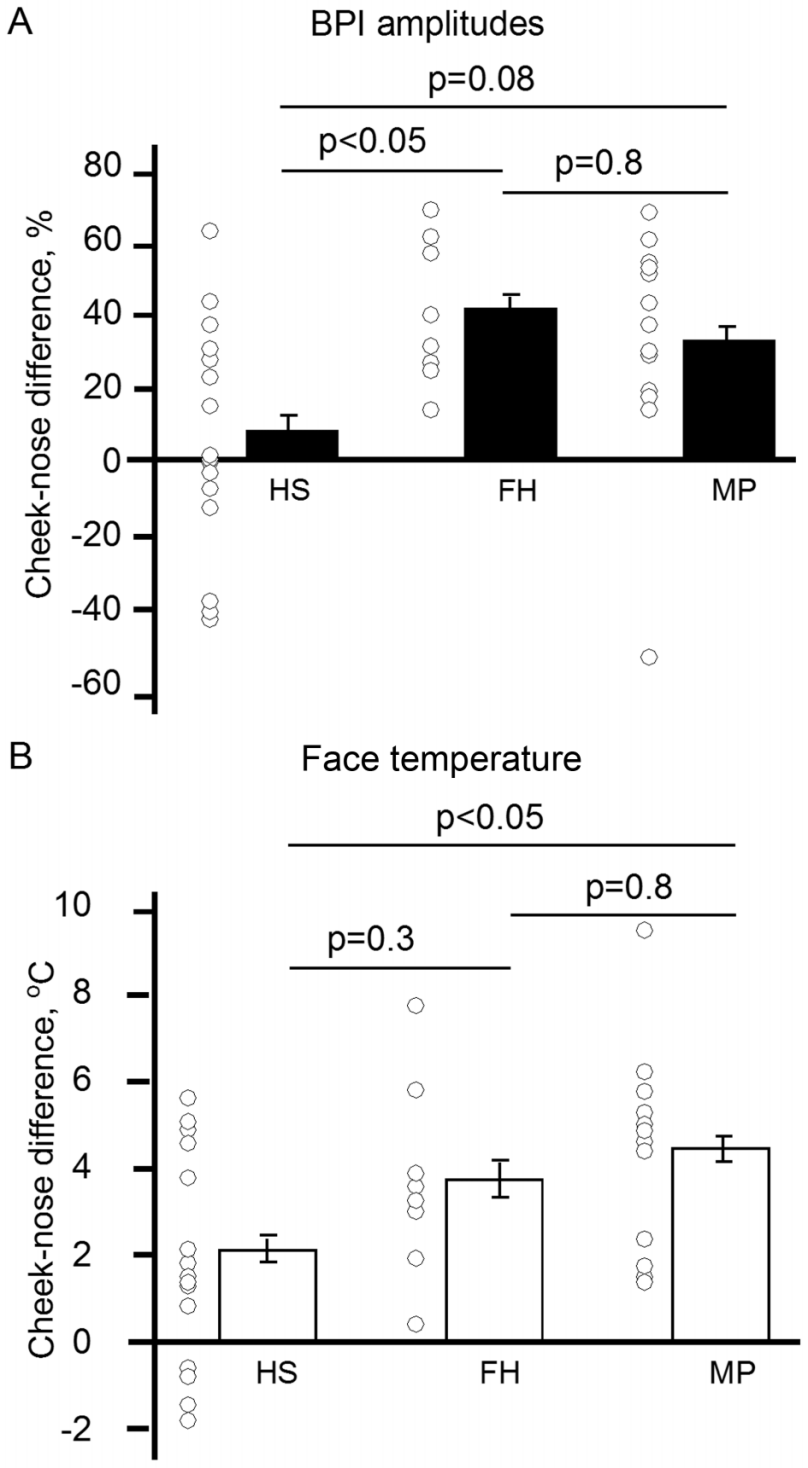
Distribution of the cheek-nose BPI amplitude difference among groups of migraine patients (MP) and healthy subjects with (FH) and without (HS) family history of migraine as measured by A: the BPI system and B, far-infrared thermography (skin temperature).

Given that the BPI method allowed a two dimensional mapping of the blood supply to the face, we searched for face regions with uneven blood perfusion (see Fig. 2). BPI mapping revealed reduced amplitude of blood pulsations in the nose area compared to cheeks in the FH and MP groups, in contrast with the HS group (p<0.05, one-way ANOVA, Tukey post-hoc test, Fig. 6A). Consistent with this finding, the higher levels of cheek-nose temperature difference (colder nose) were found in the MP group in the thermography measurements (p<0.05, one-way ANOVA), the values differed between the MP and HS groups (Tukey post-hoc test, Fig. 6B). Notably, the skin temperature was higher in the nose than in the cheek area in four healthy women only. Taken together, our data suggest that migraine is associated with lateralization of blood supply and asymmetrical blood pulsations in the face, accompanied with the signs of vasoconstriction in the nasal area.

### Factors affecting blood pulsations

**Figure 7:**
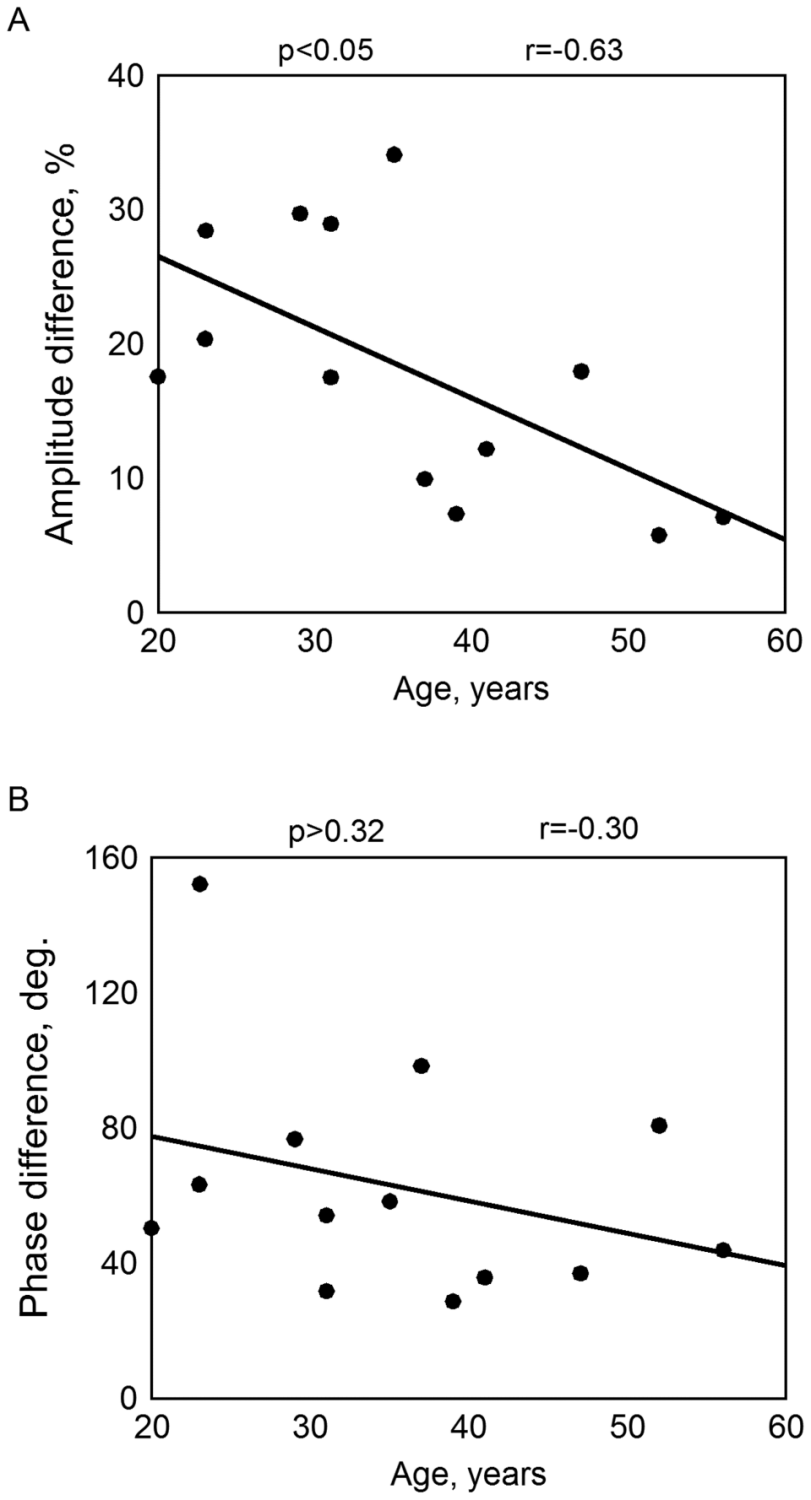
Dependence of the BPI asymmetry parameters on the subject’s age for migraineurs: A, the amplitude asymmetry; B, the temporal asymmetry.

Since the incidence of migraine is age-dependent [18], we checked the dependence of the BPI amplitude and temporal asymmetry on the age of the subjects. Figure 7A shows that there was a strong correlation of the amplitude asymmetry with the age (r  =  –0.63, p<0.05, Spearman correlation analysis) in the MP group, while the temporal asymmetry did not correlate significantly with the age of the subjects (r  =  –0.30, p > 0.32, Fig. 7B). Moreover, no correlation was found between the amplitude asymmetry and the age for healthy subjects.

**Figure 8:**
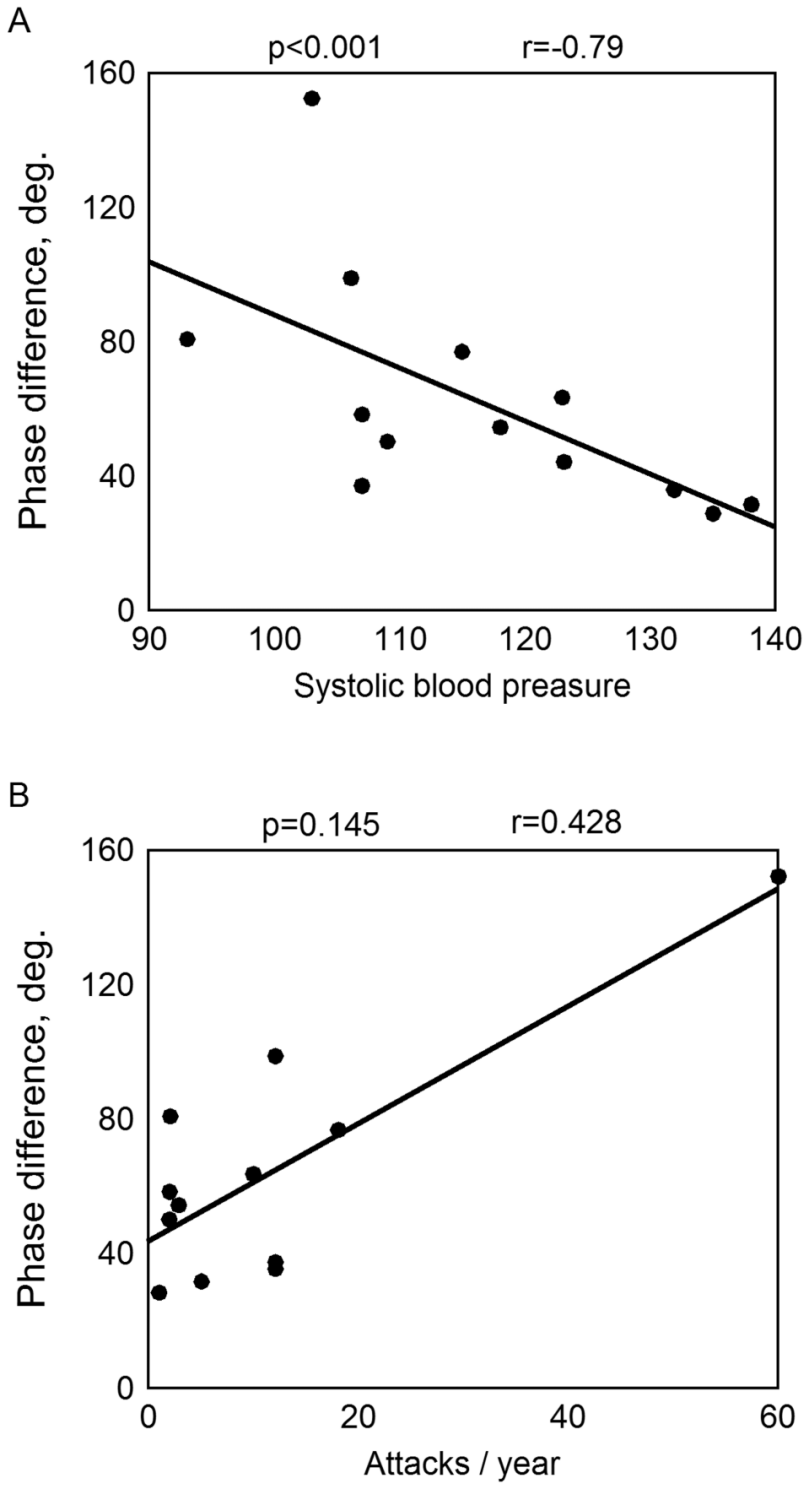
The temporal asymmetry in the MP group as a function of A, systolic blood pressure, and B, the frequency of the migraine attacks.

As shown above, the temporal asymmetry was significant for all subjects from the MP group. In this group it was inversely correlated with the blood pressure: r  =  –0.79, p<0.001 for the systolic (Spearman correlation analysis, Fig. 8A) and r  =  –0.62, p<0.05 for the diastolic pressure. Migraineurs who used anti-migraine medications demonstrated greater temporal asymmetry (p<0.05, Mann-Whitney U-test) probably due to more severe disease. The temporal asymmetry also seemed to increase with the frequency of the attacks, reaching the maximal value in subject with the highest frequency (r  =  0.43, p  =  0.145, Fig. 8B). No difference was found in BPI parameters between migraineurs with and without aura.

## Discussion

The main finding of our study was the asymmetry of blood pulsations and asynchronous blood supply in the face of migraineurs, suggesting the lateralization of the blood flow in migraine in the interictal stage. The asynchronicity of facial blood perfusion correlating with the side of unilateral headache is almost an exclusive characteristic feature of subjects suffering from migraine and could be used as a new biomarker in the diagnostics of the disease.

### Amplitude asymmetry and asynchronicity of facial blood perfusion

The new method of BPI applied in the present study allowed the measurement of two independent parameters, the pulsation amplitude and the temporal characteristics of the blood flow [14], [17]. In the MP group, an asymmetry in both independent parameters assessing the facial blood flow (the blood-pulsation amplitude and relative delay in time of blood pulsations), were observed, suggesting a strong deviation of blood perfusion from the normally synchronous blood supply to the face. Interestingly, the parameter of the temporal asymmetry demonstrated striking power to distinguish between healthy subjects and migraine patients (with a sensitivity of 100%). Remarkably, we found a strong correlation between the side of headache and the side primarily supplied by the blood after each cardiac cycle. Previously, difference between right- and left-side migraineurs has been demonstrated in the forehead photoplethysmography results [19], in cerebral blood flow characteristics [20], and in the electrodermal autonomic responses [21].

Our findings suggest that application of the BPI technique in the interictal stage can be used for fast non-invasive identification of the side of headache. In the FH group, the amplitude asymmetry of facial blood perfusion also demonstrated larger levels than in the group of HS. These findings probably indicate 'hidden' features of migraine in the FH group. Consistent with this, in the FH group, some migraine-like features have been described earlier representing a risk factor for developing of this disease [22], [23]. However, lack of clinical features of migraine or other types of primary headache in FH group suggests that the amplitude asymmetry of blood pulsations indicates less significant deviation from the normal blood supply than the temporal asymmetry, and may probably indicate a predisposition to migraine. Moreover, not all subjects of the FH group are genetically predisposed to migraine [22]. The combination of the two types of asymmetry perhaps is needed to be associated with typical migraine. Prospective cohort studies are necessary to establish if the amplitude asymmetry could be a premonitory sign of appearance of migraine in non-migraineurs with family story of migraine.

Recently, Amin et al. [24] applied magnetic resonance angiography of extra- and intracranial vessels to test potential dilatation of blood vessels during migraine attack. They detected no significant dilatation of extracranial arteries during migraine attack in pain sides. However, unlike their study which addresses the large vessels, our observations with BPI approach concerned mainly the microcirculatory blood flow in the interictal period. It is also likely that the asynchronous blood perfusion in small size vessels observed in our study can occur without essential dilatation of arteries.

### Potential mechanisms of asymmetry

Since our findings were made in the interictal period we suggest that migraine is associated with sustained lateralization of blood supply to the face even between attacks. While an amplitude asymmetry may have different explanations (and observed also in the FH group), strong temporal asymmetry exclusively observed in migraineurs clearly suggests that synchronization of the blood delivery to different parts of the subject’s head is impaired. One reason could be that asymmetry is caused by morphological asymmetrical structure of the vascular bed in migraine patients. This suggestion is consistent with the lack of correlation between age and temporal asymmetry (unlike strong correlation between age and amplitude asymmetry). Although there is a fundamental morphological asymmetry associated with the structure of aorta and other big vessels providing blood flow to the head and face, this potential reason for asymmetry is probably compensated by autonomous control over vessels since in healthy subjects a synchronous pattern of blood waves to the face was obvious. Therefore, more likely reason of the temporal asymmetry in migraineurs is essential dysfunction of the autonomic nervous system. If so, the question remains, whether observed asymmetry stems from the one-side prevalence or dysfunction of sympathetic or parasympathetic innervation. Previously, an asymmetry of autonomic regulation of blood flow in the face of migraineurs has been demonstrated by using infrared thermography [11], [12] and measurements of sympathetic skin responses [13] supporting the impairment of the sympathetic control in the face of migraine patients. Drummond explained the asymmetry by the activation of the trigeminovascular reflexes on the symptomatic side. Thus, a decrease in cervical sympathetic outflow may be secondary [12].

The idea of compromised autonomous control in migraine has already been widely discussed [25]–[27]. Our finding of lower amplitude of blood pulsations in the nasal area of migraineurs suggests a stronger activation of the sympathetic vasoconstrictory nerves [28], [29]. However, in left-side migraineurs an increased parasympathetic activation in response to the pain has been suggested [19], supporting the link between autonomic imbalance and laterality of headache. In line, asymmetrical brainstem dysfunction in migraine has been previously shown by positron emission tomography (PET) studies [30], [31]. More detailed study of spatial distribution of facial blood pulsations and other characteristics of complicated facial autonomous control [25] including the functional state of trigeminovascular system, should be carried out. Such studies could reveal the type of autonomous dysfunctions in the vascular control in migraineurs.

### Approach validated by thermography

Photoplethysmography has been reported to be a suitable method for evaluation of the pulsating blood volume [32], and it has been applied in migraine [19]. However, traditional contact methods have limitations e.g. due to measurements of the vascular function only in separate spots. Detailed mapping of the face by the BPI method allows also the finding of regions with reduced blood supply and link these with headache probability. Our face screening indicated that the nose has the lowest amplitude of blood pulsations in migraineurs. Simultaneous registration of skin temperature validated these data indicating that the nose temperature (which likely reflects the intensity of blood supply and differences in innervation to this region) [25], [28], [33] was indeed lower in migraineurs. Both methods indicated that the blood is more uniformly delivered over the face of subjects in the HS group than in the FH or MP groups. Interestingly, previously we found that subjects with family history of migraine who generate headache after sublingual nitroglycerin also showed lower nose temperature than control subjects [34]. However, the biomarker of temporal asymmetry (asynchronicity) of blood pulsations distribution shows much more pronounced difference between healthy subjects and migraineurs than the thermographic imaging.

As we showed recently, the amplitude of blood pulsations measured by the BPI system is related with the blood perfusion [17]. Indeed, the low perfusion is associated with low amplitude of blood pulsations and high perfusion can be achieved only when the pulsations of the blood are high because the blood is transported due to heart-driven hydraulic pulses [35]. The relative phase of blood pulsations quantitatively indicates any delay in the blood delivery to different areas of the body and could contain physiologically important information of blood perfusion in these areas.

The nature of the vasoconstriction behind the ‘colder nose’ finding remains unclear. In addition to local changes in vascular reactivity, migraine has been proposed to be a manifestation of a systemic vasculopathy [36]. In addition, a relatively cold nose has been associated also with the emotions [29], and migraineurs are more sensitive to stress [37]. In summary, our data are consistent with the view [38] that migraine, especially migraine with aura, represents a final common phenotype of different pathogenic mechanisms including increased risk of stroke, right-to-left shunt, potential intravascular embolism, inherited vascular defects or autonomous dysfunction.

### Limitations of the study

In the present study, the subjects in the MP group all had migraine according to the criteria of the International Classification of Headache Disorders (2004) [10]. However, subjects from the FH group, having the first degree relatives suffering from migraine, might also have some subtle signs of migraine [22], which nonetheless did not fully meet the above diagnostic criteria. Although we used blind approach for evaluation of facial asymmetry and clear differences in several studied parameters were found, the number of subjects was not very high. In addition, the severity of migraine and frequency of attacks varied widely. Patients were studied during a headache-free period, but the timing between the previous and next attack was not analysed. Evaluation of our findings in a larger sample, and also during and outside a migraine attack as well as BPI findings in other types of headaches in future studies are certainly needed.

## Conclusions

In conclusion, we demonstrate, for the first time, an asynchronous supplement of the blood to the right and left sides of the face in migraineurs in the interictal period, in sharp contrast with symmetrical and synchronous blood delivery in the face of healthy subjects. In addition, we also found asymmetry in the amplitude of blood pulsations in the face of subjects from MP and FH groups in contrast to the HS group. This asymmetry of blood supply in the facial area could be associated with the dysfunction of the autonomic vascular control in the face of migraineurs. These new biomarkers of migraine are easily measurable and have the potential to be applied in the studies of vascular processes accompanying migraine/headache. Study findings may further develop our understanding of migraine pathophysiology.

## Supporting Information

Video S1 **Dynamics of the blood volume pulsation during single cardiac cycle for subject suffering from migraine.** For better visualization the speed of video was slowed down.
(M4V)

Video S2 **Dynamics of the blood volume pulsation during single cardiac cycle for healthy woman.** For better visualization the speed of video was slowed down.
(M4V)
